# Treatment of bone metastases from solid tumors with bone-modifying agents: a web survey of Italian oncologists investigating patterns of practice drug prescription and prevention of side effects

**DOI:** 10.1007/s00520-024-08392-8

**Published:** 2024-03-01

**Authors:** Vittorio Fusco, Massimo Di Maio, Anna Amela Valsecchi, Daniele Santini, Marcello Tucci, Ugo De Giorgi, Paolo Bossi, Toni Ibrahim, Luigi Cavanna, Gaetano Lanzetta, Maura Rossi, Giorgia Rossetti, Mario Airoldi, Alessandro Comandone, Saverio Cinieri

**Affiliations:** 1 Oncology Unit, Department of Medicine, Azienda Ospedaliera-Universitaria “SS Antonio E Biagio e Cesare Arrigo”, Alessandria, Italy; 2 Translational Medicine Unit, DAIRI Department of Integration, Research and Innovation, Azienda Ospedaliera-Universitaria “SS Antonio E Biagio e Cesare Arrigo”, Alessandria, Italy; 3 Department of Oncology, University of Turin, Ordine Mauriziano Hospital, Turin, Italy; 4 UOC Oncologia A, Policlinico Umberto 1, La Sapienza Università, Rome, Italy; 5 Department of Medical Oncology, Cardinal Massaia Hospital, Asti, Italy; 6 Department of Medical Oncology, IRCCS Istituto Romagnolo Per Lo Studio Dei Tumori (IRST) “Dino Amadori”, Meldola, Italy; 7 Medical Oncology, Humanitas Cancer Center, Milan, Italy; 8 Osteoncology, Bone and Soft Tissue Sarcomas and Innovative Therapies Unit, IRCCS Istituto Ortopedico Rizzoli, Bologna, Italy; 9 Medicine and Oncology Area, Clinica Piacenza” Hospital, Piacenza, Italy; 10 Medical Oncology Unit, Italian Neuro-Traumatology Institute, Grottaferrata, Italy; 11 Head Office, Rete Oncologica Di Piemonte E Valle d’Aosta, Turin, Italy; 12 Medical Oncology Unit, Ospedale Di Summa A. Perrino, Brindisi, Italy; 13 Italian Network for Supportive Care in Cancer (NICSO), Milan, Italy; 14 Italian Society of Osteoncology (ISO), Bologna, Italy; 15 Collegio Italiano Dei Primari Oncologi Medici Ospedalieri (CIPOMO), Genoa, Italy; 16 AIOM Guidelines Working Group, Grottaferrata, Italy

**Keywords:** Bone metastases, Bone-modifying agents, Bisphosphonates, Denosumab, Survey, Osteonecrosis

## Abstract

**Purpose:**

Optimal use of bone-modifying agents (BMAs) in patients with bone metastases from solid tumors is uncertain in some aspects: the drug choice; the planned treatment duration and long-term therapy; the prevention and management of possible side effects, including renal toxicity, hypocalcaemia, and medication-related osteonecrosis of the jaw (MRONJ).

**Methods:**

Italian oncologists were invited to fulfil a 24-question web survey about prescription of BMAs for bone metastases of breast cancer, prostate cancer, and other solid tumors. Prevention and management of side effects were also investigated.

**Results:**

Answers of 191 oncologists were collected. BMAs are usually prescribed at the time of diagnosis of bone metastases by 87.0% (breast cancer) and 76.1% (solid tumors except breast and prostate cancers) of oncologists; the decision is more articulated for prostate cancer (endocrine-sensitive versus castration-resistant). The creatinine level (32.3%), the availability of patient venous access (15.8%), and the type of primary neoplasm (13.6%) are the most reported factors involved in choice between bisphosphonates and denosumab. Zoledronic acid every 3 months was considered as a valid alternative to monthly administration by 94% of Italian oncologists. Oncologists reported a good confidence with measures aimed to prevent MRONJ, whereas uncertainness about prevention and management of hypocalcemia was registered.

**Conclusion:**

Italian oncologists showed a high attitude in prescribing bisphosphonates or denosumab at the time of diagnosis of bone metastases, with a large application of preventive measures of side effects. Further studies are needed to investigate some controversial aspects, such as optimal drug treatment duration and long-term drug schedules.

## Introduction

Bone is a frequent site of metastases in patients with solid tumors (breast, prostate, lung, renal, and other cancers) [1]. Bone-modifying agents (BMAs), also known as bone-targeted agents or antiresorptive drugs, include bisphosphonates and denosumab; they are largely recommended and administered to prevent skeletal-related events (SREs) in patients with cancer metastatic to the bone [2].

International recommendations and guidelines were delivered regarding BMA treatment in bone metastatic cancer disease and the management of BMA side effects [3–7]. In Italy, national guidelines are periodically published in Italian language by AIOM (Italian Association of Medical Oncology) on the treatment of bone metastases and care of bone health in cancer patients [8]. Detailed recommendations for the prevention and management of medication-related osteonecrosis of the jaw (MRONJ), the main side effect of BMAs, were released by the Italian Societies of Maxillofacial Surgery (SICMF) and Oral Medicine (SIPMO) and endorsed by AIOM and other Italian scientific bodies [9].

Guidelines and recommendations show some heterogeneity and uncertainty about the choice of treatment drug (bisphosphonates versus denosumab), optimal treatment duration (one versus 2 years or indefinite treatment), de-escalation and delayed dosing schedules (e.g., zoledronic acid every 3 months instead of monthly administration) [3–5, 8, 10], as well as the management of side effects, such as MRONJ and hypocalcemia [6–9, 11]. According to recent surveys and studies conducted in Europe and North America, the attitudes of prescribing physicians, as well as patients’ preferences and real-life practice patterns, are not fully homogeneous [12–28].

In November 2020, AIOM launched a national survey in Italy based on a 24 items questionnaire form previously used for internal investigation by the *Rete Oncologica di Piemonte e Valle d’Aost*a, a cancer network in North-Western Italy, to investigate the BMA prescription attitude and the side effect management patterns among Italian oncologists.

## Methods

Between November 2020 and January 2021, Italian oncologists who were AIOM members, were invited by e-mail to participate in an anonymous web survey concerning the prescription of BMAs, including bisphosphonates and denosumab, in the treatment of bone metastases of solid tumors, and the management of related side effects.

The invitation was also endorsed by the Italian College of Hospital Medical Oncology Unit Directors (CIPOMO), the Italian Society of Osteoncology (ISO), the Italian Network for Cancer Supportive Care (NICSO—the Italian branch of the Multinational Association of Supportive Care in Cancer, MASCC), and the Rete Oncologica di Piemonte e Valle d’Aosta, to include a large number of oncologists in the survey.

Answers to questions were anonymously provided on a voluntary basis, and data was collected in compliance with national and EU regulations on the protection of the processing of personal and sensitive data (European Regulation n.679/2016, and Italian data protection legislation).

The Italian language questionnaire consisted of 24 questions and was divided into two parts. The first part (6 questions) collected personal data of oncologists: gender, age (< 40; 40–50; 50–60; > 60 years), region of residence (North-Western Italy, North-Eastern Italy, Central Italy, Southern Italy plus Sicily and Sardinia), workplace (general hospital, university hospital, cancer center, private clinic, or other institution), role (hospital oncologist, university oncologist, resident/trainee, private practitioner), and affiliation to the cancer societies or organizations cited above. The second part (18 questions) aimed at evaluating the attitude and the time of prescription of BMAs in patients with breast cancer, prostate cancer, and other bone metastatic cancers (lung, renal, etc.); the choice of drug (bisphosphonate versus denosumab); the dental evaluation before start of BMA therapy; the routine blood test before BMA drug administration; the calcium and vitamin D supplementation; and the management of hypocalcemia. Most questions were closed with either single or multiple answers permitted.

## Results

Out of 2248 invited oncologists, 191 responded (response rate 8.5%). Respondents’ characteristics are shown in Table 1. Females were 51.3% and males 48.7%. 37.2% were younger than 40 years; 15.7% were between 40 and 50; 30.9% were between 50 and 60, and 16.2% older than 60. 42.9% worked in North-Western regions of Italy; the others were equally divided between North-Eastern, Central Italy, Southern Italy plus Islands. Most of the participants (54.9%) worked in general hospitals, 22.5% in university hospitals, and 16.8% in research cancer centers. A large majority (78%) were hospital specialists; 9.9% were university affiliates; 8.9% were trainees, and 3.2% worked in private institutions. Of the 191 oncologists, 125 were registered only with AIOM, while another 66 were also registered with other societies or organizations (NICSO/MASCC, 18; ISO, 7; CIPOMO, 16; other 25). The answers to the second part of the survey are reported in Table 2.

**Table 1:** Oncologists’ characteristics

*Feature*	*Options*	*Number (percentage)*
Sex	Male	93 (48.7%)
	Female	98 (51.3%)
Age	Less than 40 years	71 (37.2%)
	Between 40 and 50 years	30 (15.7%)
	Between 50 and 60 years	59 (30.9%)
	More than 60 years	31 (16.2%)
Region of workplace	North-Western Italy	82 (42.9%)
	North-Eastern Italy	37 (19.5%)
	Central Italy	36 (18.8%)
	Southern Italy and Islands (Sicily and Sardinia)	36 (18.8%)
Health organization	General Hospital	105 (54.9%)
	University Hospital	43 (22.5%)
	IRCCS (Italian Cancer Center)	32 (16.8%)
	Other	11 (5.8%)
Medical role	Hospital oncologist	149 (78.0%)
	University oncologist	19 (9.9%)
	Resident/trainee	17 (8.9%)
	Private practitioner	6 (3.2%)
Membership	Only AIOM member	125 (65.4%)
	AIOM and other society/organization member	66 (34.6%)

**Table 2:** Summary of answers to the second part of the survey

*Questions and options*	*Number (percentage)*
You prescribe antiresorptive drugs (bisphosphonates, denosumab) for patients with bone metastases from breast cancer:	
a) Always, at the time of diagnosis of bone metastases b) Almost always (except for a minority of patients) c) Only in symptomatic cases or in cases at high risk of skeletal-related events (SREs) d) Rarely e) Never f) I do not follow patients with breast cancer	60 (37.1%) 81 (50%) 20 (12.3%) 1 (0.6%) 0 (0%) 29
You prescribe antiresorptive drugs (bisphosphonates, denosumab) for patients with bone metastases from prostatic carcinoma:	
a) Always, at the diagnosis of bone metastases, regardless of hormonal responsiveness b) Only in castration-resistant carcinoma, regardless of symptomatology c) Only in castration-resistant carcinoma, and only in symptomatic cases or which I consider to be at high risk of SRE d) In both hormone-dependent and castration-resistant carcinoma, if symptomatic e) Never f) I do not follow patients with prostate cancer	53 (37.9%) 46 (32.8%) 29 (20.7%) 12 (8.6%) 0 (0%) 51
You prescribe antiresorptive drugs (bisphosphonates and denosumab) for patients with bone metastases from other solid tumors (lung, renal cancer, etc.):	
a) Always, at the time of diagnosis of bone metastases b) Almost always (except for a minority of patients) c) Only in cases of symptomatic cases or at high risk of skeletal events (SRE) d) Rarely e) Never f) I do not follow this type of patient, except occasionally	35 (19.9%) 79 (44.9%) 55 (31.2%) 7 (4%) 0 (0%) 15
You ask for dental evaluation before prescription of antiresorptive drugs (bisphosphonates, denosumab)	
a) Always, systematically b) Almost always c) Only in case of poor oral hygiene of the patient d) Rarely e) Never	173 (90.6%) 17 (8.9%) 1 (0.5%) 0 (0%) 0 (0%)
The pre-therapy dental evaluation usually includes:	
a) Dental panoramic X-rays and dental examination b) Only dental panoramic X-rays c) Only dental examination	183 (95.8%) 2 (1.1%) 6 (3.1%)
For most of your patients, pre-therapy dental evaluation takes place:	
a) Entirely at my hospital b) Entirely in another structure (upon reservation/organization by my center staff) c) Partly internal, partly external (e.g., dental panoramic X-rays in a public structure and dental visit at a private practice) d) All delivered to individual choice and/or personal initiative of the single patient	90 (47.1%) 23 (12.1%) 54 (28.2%) 24 (12.6%)
If one or more dental extractions are programmed by the dentist:	
a) You always start therapy immediately and delay the tooth/teeth extraction b) You always wait for extraction before starting the treatment c) You start the treatment in selected cases (e.g., aggressive disease) and wait for extraction in most cases d) You start immediately in most cases and wait for extraction only in selected cases (e.g., asymptomatic disease with a good prognosis)	2 (1.1%) 125 (65.4%) 58 (30.4%) 6 (3.1%)
In case of waiting after extraction, you usually start the treatment:	
a) 2 weeks from extraction, and after dental check-up (closed cavity) b) After 4 weeks from extraction, and after dental check-up (closed cavity) c) 4 weeks after extraction, regardless of dental check-up d) After 6–8 weeks, regardless of dental check-up e) Other	34 (17.8%) 119 (62.3%) 11 (5.7%) 25 (13.1%) 2 (1.1%)
You prescribe for patients with bone metastases from solid tumors:	
a) Always zoledronic acid b) Always bisphosphonates (zoledronic acid, ibandronate, or pamidronate, depending on the patient) c) Always denosumab d) Zoledronic acid or denosumab, depending on the patient e) Other	22 (11.5%) 5 (2.6%) 14 (7.3%) 150 (78.6%) 0 (0%)
Are there limitations to denosumab prescription (in addition to the need to fill out the AIFA – the Italian Medicine Agency—form) in your center?	
a) No b) Yes, by hospital commitment or choice c) Yes, due to my workgroup indications d) Other	163 (85.3%) 9 (4.7%) 16 (8.4%) 3 (1.6%)
If you prescribe zoledronate or denosumab on the individual patient, the choice depends on:	
*Select maximum 3 possible answers* Primary cancer type Prognosis of the cancer patient Symptomatology Age of the patient Risk of skeletal-related events (SREs) Blood creatinine level Calcium level Availability of venous access Patient oral health Other	57 29 18 37 24 135 37 66 5 9
During treatment with zoledronic acid and other bisphosphonates, you check:	
a) Only creatinine level, periodically b) Only calcium level, periodically c) Creatinine and calcium level, periodically d) Only creatinine level, before each infusion e) Only calcium, before each infusion f) Creatinine and calcium, before each infusion e) Other	0 (0%) 0 (0%) 30 (15.7%) 3 (1.6%) 1 (0.5%) 155 (81.1%) 2 (1.1%)
During treatment with denosumab, you check:	
a) Only creatinine level, periodically b) Only calcium level, periodically c) Creatinine and calcium level, periodically d) Only creatinine level, before each administration e) Only calcium, before each administration f) Creatinine and calcium, before each administration e) Other	0 (0%) 4 (2.0%) 27 (14.1%) 2 (1.1%) 26 (13.6%) 125 (65.5%) 7 (3.7%)
Did you find hypocalcemia after denosumab or bisphosphonates?	
a) No b) Sporadically, and always asymptomatic c) Sporadically, with few symptomatic cases d) Quite frequently, but always asymptomatic e) Quite frequently, and with some symptomatic cases	0 (0%) 119 (62.3%) 54 (28.3%) 13 (6.8%) 5 (2.6%)
How do you usually treat asymptomatic hypocalcemia?	
a) I temporarily interrupt the treatment and check the calcium later b) I prescribe oral calcium c) I prescribe calcium by intravenous infusion d) I prescribe high doses of vitamin D e) Other	32 (16.8%) 108 (56.6%) 16 (8.4%) 15 (7.8%) 20 (10.4%)
In case of treatment with bisphosphonates, as a supplementation you prescribe in most patients:	
a) Only calcium b) Only vitamin D c) Calcium and vitamin D separately d) Calcium + vitamin D associations f) Nothing	2 (1.1%) 11 (5.7%) 60 (31.4%) 112 (58.6%) 6 (3.2%)
In case of treatment with denosumab, as a prophylaxis you prescribe in most of patients:	
a) Only calcium b) Only vitamin D c) Calcium and vitamin D separately d) Calcium + vitamin D associations e) Nothing	4 (2.1%) 10 (5.2%) 57 (29.8%) 114 (59.7%) 6 (3.2%)
What do you think about a quarterly administration of zoledronic acid?	
a) I am not convinced that it can be a reliable alternative to the monthly administration of zoledronic acid b) It can be a valid alternative to monthly administration, after one year of monthly infusions c) It is a valid “upfront” alternative (from the beginning of therapy) to monthly administration, in some patients (e.g., pauci-symptomatic patients, or with mildly aggressive disease, etc.) d) Other	9 (4.7%) 107 (56.0%) 72 (37.7%) 3 (1.6%)

### Attitudes toward early or delayed BMA prescription

The BMA time prescription attitudes of oncologists in case of bone metastases from breast cancer versus prostate cancer versus other solid tumors were investigated.

Among the 162 oncologists who follow patients with breast cancer, 60 (37.0%) reported to prescribe antiresorptive drugs (bisphosphonates and /or denosumab) “always, at the time of diagnosis of bone metastases,” 81 (50.0%) “almost always (except for a minority of patients),” and 20 (12.3%) “only in symptomatic cases or in cases at high risk of skeletal-related event (SREs).”

For patients with bone metastases from prostate carcinoma, 53 (37.8%) out of 140 BMA prescribers stated that they administer antiresorptive drugs “always, at the diagnosis of metastases, regardless of hormone responsiveness,” whereas 75 (53.6%) oncologists declared to prescribe BMAs only in castration-resistant cases: more specifically 46 (32.8%) regardless of the symptoms and 29 (20.7%) only in symptomatic cases or considered to be at high risk of SREs. Other 12 oncologists (8.6%) declared to prescribe BMAs both in hormone-dependent and castration-resistant prostate cancer, if symptomatic.

Patients with bone metastases from solid tumors other than breast and prostate were followed by 176 oncologists, who gave the following answers: 35 (19.9%) declared to prescribe BMAs “always, at the diagnosis of bone metastases”; 79 (44.9%) “almost always, except for a minority of patients”; 55 (31.2%) only in symptomatic cases or patients evaluated as at high risk of SREs; and 7 (3.9%) rarely.

### Choice of drug and schedule

Regarding the type of antiresorptive drug used in patients with bone metastases, 150 (78.5%) of oncologists reported that they prescribe zoledronic acid or denosumab depending on characteristics of each patient, while 22 (11.5%) always prescribe zoledronic acid, and 14 (7.3%) always prescribe denosumab. Only 5 (2.6%) choose between various bisphosphonates (zoledronic acid, ibandronate, or pamidronate) on a case-by-case basis.

One hundred sixty-three (85.3%) oncologists reported that they had no limitations about the prescription of denosumab (in addition to the need of filling out the national registry case report form, requested by AIFA—the Italian Medicine Agency), while the remaining oncologists could not usually prescribe denosumab due to their hospital recommendations (4.7%), working group guidelines (8.3%), or other reasons (1.5%).

The more important criteria of choice of drug for each patient were the patient level of blood creatinine (32.3%), the availability of patient venous access (15.8%), and the type of primary neoplasm (13.6%). Finally, albeit in lower percentages, other factors that influence the therapeutic choice were cancer prognosis (6.9%), symptoms (4.3%), patient age (8.8%), expected risk of SRE (5.7%), patient calcium level (8.8%), and patient oral health (1.1%).

One specific question addressed the opinion of oncologists about possible administration of zoledronic acid every 3 months. Out of 191 oncologists, 107 (56%) considered the quarterly administration as “a valid alternative to the continuous monthly administration, after one year of monthly infusions,” 72 (37.7%) stated it is “a valid ‘upfront’ (from the beginning of therapy) alternative to monthly administration, in some patients (e.g., pauci-symptomatic patients, or with mildly aggressive disease, etc.)”, 9 oncologists (4.7%) did not consider quarterly zoledronic acid as a reliable treatment, and 3 expressed other evaluations.

### Calcium and vitamin D supplementation

Calcium and vitamin D (as single drugs or together in associated forms) were prescribed by about 90% of oncologists in case of bisphosphonate or denosumab treatment, whereas 7% prescribed only calcium or vitamin D, and 3% did not prescribe supplementation.

### Patient workout before single BMA administration

During treatment with zoledronic acid and other bisphosphonates, 81.5% of oncologists reported to check the blood level of creatinine and calcium before each single infusion and 15.7% periodically; 3 oncologists (1.5%) reported to check creatinine level only.

In case of treatment with denosumab, they reported to check the blood level of creatinine and calcium before each administration (65.4%) or periodically (14.1%), while another 13.6% reported to test only the calcium level before each administration.

### Hypocalcemia

One hundred nineteen out of 191 oncologists (62.3%) reported that they sporadically found hypocalcaemia after denosumab or bisphosphonates and always asymptomatic, 54 (28.3%) sporadically but with some symptomatic cases, and 13 (6.8%) not infrequently but always asymptomatic. Only 5 oncologists reported to have encountered hypocalcemia frequently, including some cases with symptoms.

Oncologists were asked about their pattern of treatment of asymptomatic hypocalcemia (one possible answer allowed). Most of them reported to prescribe oral calcium (56.5%) or intravenous calcium (8.4%) or high doses of vitamin D (7.8%). Temporary drug suspension and further calcium check were indicated as the main option by 32 (16.7%) and “other” (reporting a combination of measures) by 20 (10.4%).

### Preventive dental evaluation

One hundred seventy-three out of 191 (90.5%) oncologists reported to require always and systematically a dental evaluation before starting BMAs for metastatic bone cancer patients. The evaluation included both dental panoramic X-ray (RX) and dental examination in 95.8% of cases. They stated that pre-therapy dental evaluation can take place in the same hospital of the oncology unit (47.1%), or partially inside and partially outside the hospital (e.g., RX examination in a public hospital and dental visit at a private practice) (28.2%); 12% of prescribers entrusted the assessment to another public structure (upon reservation/organization by the oncology unit staff). The remaining 12.5% left (by choice or necessity) the dental evaluation to the patient’s initiative.

Oncologists were asked for their attitude in case the dentist plans one or more preventive tooth extractions before BMA starts. 65.4% of oncologists reported to always wait for the extraction before starting treatment; 30.4% stated to start the treatment in selected cases (e.g., aggressive metastatic disease), while waiting for the extraction in most of cases, and 3.1% to start immediately the BMA therapy in most cases and delay the extraction in selected cases (e.g., asymptomatic disease with a good prognosis). 1.1% of oncologists routinely started the antiresorptive therapy at once, delaying the dental care.

After extraction, 62.3% of oncologists reported to start the drug treatment not less than 4 weeks after the extraction and only after dental check-up for healing (closed alveolus), 17.8% to wait less than 4 weeks (but after dental check-up), and 18.7% to wait 4 weeks or more, regardless a further dental check-up.

## Discussion

BMAs have a relevant role in the management and supportive care of patients with bone metastases from solid cancer. Although no impact on survival was demonstrated, several trials showed that BMAs reduce the risk of SREs, including bone pathological fractures, need of surgery or radiation to the bone, and spinal cord compression, and are largely recommended in this setting [3–5, 8]. Recently, symptomatic skeletal events (SSEs), including symptomatic bone pathological fractures, bone surgery, bone palliative radiation, and symptomatic spinal cord compression, have been considered to perform better than SREs as evaluation criteria [29]. Start of BMA therapy is generally recommended early after diagnosis of bone metastases to prevent or delay SREs [3–5, 8], and it is frequently reported within the first 3 months [12, 13, 25, 27].

The optimal use of BMAs in patients with bone metastases is still uncertain in several aspects, above all in daily clinical practice, as BMA trials did not clarify this topic [10, 30–32].

The drug choice may depend on many criteria: direct and indirect costs, real or supposed risk of SREs or SSEs, risk of early disease progression and/or worsening of performance status or quality of life (QoL), comorbidities, risk of side effects, patient’s preferences, and life expectancy [10, 16, 17, 19, 20, 23–25, 27, 30–32].

The drug cost (for individuals or healthcare systems) and several indirect costs (e.g., hospital facilities, staff for intravenous versus subcutaneous drug administration, costs for blood calcium and creatinine monitoring, dental check-ups, etc.) are surely important [10, 30–32]. Large differences in cost are linked to drug reimbursement or availability rules, regional-country specificity, and type of healthcare system [12, 17, 19–21, 23].

In most trials, BMAs were administered for a maximum of 2 years, and there are no uniform recommendations about the length of initial planned BMA treatment duration [3–5, 10]. Several surveys [14, 16, 19, 23] have registered oncologists planning monthly BMAs (bisphosphonates or denosumab) for 1 or 2 years, or indefinitely (i.e., to deterioration of performance status, as specified by less recent guidelines) [33].

The attitude after the first 1 or first 2 years of BMA treatment can be very different, with temporary stop or de-escalation (to quarterly treatments, with the same drug or shifting from a drug to another), representing a challenging choice for oncologists [3–5, 10, 14, 16, 19, 23, 30–32]. A systematic review on the risk–benefit of BMA administration for more than 2 years in breast cancer and castration-resistant prostate cancer [34] concluded that evidence about BMA administration beyond 2 years is heterogeneous and is based on retrospective analysis. However, new data are emerging in favor of de-escalation [35].

The prevention and management of possible side effects of BMAs, such as MRONJ, renal toxicity, and hypocalcaemia, show some problematic issues.

Definition of MRONJ is controversial [36], with consequences in early diagnosis, staging, and appropriate treatment of the jawbone disease [6, 8–10, 37–39], as well as in evaluation of frequency data [40]. The incidence of MRONJ in patients with bone metastases receiving BMAs ranges between 1% and more than 20%, with a risk of up to 30% or higher among long-surviving subsets of advanced cancer patients [40].

Renal impairment of patient clearly favors denosumab in comparison with bisphosphonates [3].

Hypocalcaemia in patients receiving BMAs is often mild and transient, but it is to be prevented and managed because sometimes it can be serious [7, 11]. Calcium and vitamin D supplementation is systematically recommended together with BMA administration [3–5, 8, 11], but there are differences in its application. [20]

Our survey on the opinions of Italian oncologists adopted a series of questions previously used by the *Rete Oncologica di Piemonte e Valle d’Aosta* (an oncology network in North-Western Italy) for internal investigation in 2015 and 2018 (unpublished results) that guided the network to formulate a consensus document [41] answering a PICO (population, intervention, comparison, outcome) question about the possible tailoring of BMA treatment in patients with bone metastases of solid cancers.

Most oncologists declared their attitude toward the early start of BMA treatment after diagnosis of bone metastases, in concordance with current recommendations [3–5, 8] and several surveys and clinical patterns studies [12, 13, 20, 25, 35]. A partial exception was for bone metastatic prostate cancer, reflecting the differences between hormone-sensitive and castration-resistant prostate tumors [42]. In our survey the question about prostate cancer did not distinguish between BMAs at high doses and with monthly administration (as usual in metastatic castration-resistant disease) or BMAs at low doses and/or with delayed administration (recommended for hormone-sensitive metastatic cancer, as well as for prevention or treatment of cancer treatment-induced bone loss (CTIBL)). This issue should be further investigated in a more specific survey involving both urologists and oncologists, as well as specialists treating CTIBL [13, 17, 20, 25, 28, 42].

Only a minority of oncologists affirmed to usually choose the same drug (11.5% always prescribing zoledronic acid, and 7.3% always prescribing denosumab) as a “one-fits-all” attitude, whereas a large majority (78.5%) affirmed to choose between zoledronic acid or denosumab case-by-case, and 2.6% stated to choose among bisphosphonates. It is to be noted that denosumab is an option without any limitations for 85.3% of the oncologists of this survey, despite higher drug cost for the Italian healthcare system in comparison with zoledronic acid and other bisphosphonates [41], counterbalanced by lower costs of administration [41].

Italian oncologists were asked for which criteria they adopted in tailoring the choice of the BMA drug in single patients. How do the more frequently cited criteria could change the drug choice? We can only argue that some factors might favor denosumab, such as a high patient level of blood creatinine (32.3%), a poor availability of patient venous access (15.8%), and a more symptomatic and aggressive cancer with a high risk of SRE in short term. Vice versa, zoledronic acid might appear more suitable in patients with less aggressive disease and with an expected high risk of hypocalcaemia and MRONJ (usually more frequent in patients receiving denosumab) [3, 11, 40]. High propensity of Italian oncologists toward a tailored choice of BMA treatment is partially confirmed by available single patient data collections in Italy, even though results are not homogeneous [14, 27, 43].

In our survey, there were no direct questions regarding favorite planned initial duration of BMA therapy (1 year versus 2 years versus indefinite treatment) or about choices on long-term therapy (e.g., interruption versus de-escalation versus indefinite monthly treatment). However, Italian oncologists were asked for possible administration of zoledronic acid every 3 months, with three alternative answers. More than one third of oncologists (72, 37.7%) considers quarterly administration as a possible choice for upfront treatment, whereas about half of them (107, 56%) considered it as a valid alternative to the continuous monthly administration, after the first year of the quarterly administration (as reported by some of trials investigating de-escalation) [3, 10, 44], and it was refused by 9 oncologists (4.7%).

Supplementation of calcium and vitamin D (as single drugs or together in associated forms) were both prescribed by 90% of oncologists, in concordance with drug prescription instructions and guidelines [3, 8].

The blood level of both creatinine and calcium was checked before each single infusion of zoledronic acid by 81.5% of oncologists, and by only 65.4% before each single subcutaneous injection of denosumab. Before denosumab administration, 14.1% reported to check creatinine and calcium levels periodically, and 13.6% of oncologists tested only the calcium level.

Hypocalcaemia was not perceived as a relevant problem by the survey respondents, probably because most events were mild and transient. Oral calcium and/or delay of BMA administration are preferred to intravenous calcium and further vitamin D supplementation, in case of asymptomatic hypocalcemia, in partial concordance with recommendations [7, 40].

MRONJ prevention is an important issue for Italian oncologists, as the Italian healthcare system strongly recommends all the measures to reduce the MRON risk in patients with bone metastatic cancer and myeloma [9]. A dental evaluation (including both dental panoramic X-rays and dental examination in 95.8% of cases) is reported as systematically adopted before starting high-dose BMA treatment by the large majority (90.5%) of oncologists, in accordance with national and international guidelines [3, 6, 8, 9]. We investigated where preventive dental panoramic X-rays and dental visits are usually performed, because unfortunately, the national Italian healthcare system covers all medical treatments for cancer patients, but not free dental care everywhere. The survey shows the difficulties of Italian oncologists to obtain a complete and rapid free dental check before BMA starts (usually easier in academic and large hospitals; very difficult in other centers): according to this survey, more than 40% of oncologists see their patients pay (out of pocket) dental visits in private practice.

In case the dental specialist (dentist or maxillofacial surgeon) recommends preventive tooth extraction(s) before the start of BMA treatment, a very high proportion of oncologists wait until extraction before starting treatment: always (65.4%) or in most of cases, excluding only cases with very aggressive metastatic cancer disease (30.3%). In case of tooth extraction, it is advisable to start the BMA treatment not too quickly, due to risk of jawbone healing impairment by bone turnover inhibition induced by BMA, with a consequent risk of early MRONJ onset [6, 9]. Most oncologists reported to start treatment not less than 4 weeks after the extraction and only after dental check-up for alveolus healing (62.3%), or to wait 4 weeks or more regardless a further dental check-up (18.7%). Another 17.8% stated to wait less than 4 weeks, but after dental check-up.

The present survey has both strengths and limitations, potentially influencing the interpretation of the results.

One point of strength is that the survey is a snapshot of recent attitudes of oncologists in all regions in Italy, both in university hospitals, cancer centers, and in general, hospitals, with an expected generalizability of the findings. The fact that some questions tried to investigate separately the usual patterns of care among oncologists treating the different types of cancer with bone metastases (breast cancer versus prostate cancer versus other solid tumors) is also a strength of the survey.

A first limitation is the small sample of respondents, possibly introducing a selective response bias. A second one is that the reported attitude of oncologists may not coincide with the daily real-world patterns of care. The coincidence or not could be registered only by independent large studies with single patient data in populations representative of all Italian cancer patients receiving BMA treatment, including large and small oncology units and all the Italian regions.

## Conclusions

Italian oncologists show a good propensity to include BMAs in treatment of patients with cancer, shortly after diagnosis of bone metastases. Oncologists appear to be aware of possible side effects and of the need of preventive measures.

Optimal initial drug treatment duration and long-term drug schedules remain controversial, and tailored BMA treatment remains an option. Further studies are needed to investigate the best practice in different categories of cancer patient.

## Data Availability

Data are available after request to V.F. (fusco.dott.vittorio@gmail.com).
